# The clinical application of Filmarray respiratory panel in children especially with severe respiratory tract infections

**DOI:** 10.1186/s12879-021-05900-7

**Published:** 2021-02-27

**Authors:** Fen Pan, Bingjie Wang, Hong Zhang, Yingying Shi, Qi Xu

**Affiliations:** Department of clinical laboratory, Shanghai Children’s Hospital, Shanghai Jiaotong University, Luding Road 355, Putuo District, Shanghai, 200062 China

**Keywords:** Filmarray respiratory panel, Severe respiratory tract infections, Respiratory virus, Children

## Abstract

**Background:**

Respiratory tract infections (RTIs) are the common diseases in children and the routine detection methods frequently fail to identify the infectious pathogens especially for viruses. The Filmarray respiratory panel (FARP) can reliably and rapidly identify viruses and bacteria pathogens. This study is to evaluate the performance and clinical significance of FARP in children.

**Methods:**

Children diagnosed with RTIs in pediatric intensive care unit (PICU) were enrolled in this study. Nasopharyngeal secretion (NPS) samples of these children were collected and the FARP assay for 17 pathogens and routine microbiological methods were performed. Clinical data of all patients was also collected and evaluated.

**Results:**

A total of 90 children were enrolled into this study and 58 patients (64.4%) were positive for 13 pathogens by FARP, with 18 being detected positive with multiple-virus (31.3%, 18/58). Human rhinovirus/enterovirus (21.0%%, 17/58) were the predominant pathogen, followed by adenovirus (18.5%). Higher proportions of various pathogens were identified in the infant and toddler (0–2 years) groups with human rhinovirus/enterovirus being mostly virus. Adenovirus were common in the group aged 3–5 years, but only three pathogens including *M.pneumoniae*, respiratory syncytial virus, and adenovirus were also found in age group (6–14 years). Among 58 FARP positive patients, significant differences were found in antibiotic prescription and use of glucocorticoid between the single-organism-positive group and the multi-organism-positive group (*P* < 0.05). Furthermore, there was significant difference in use of anti-virus and usage of glucocorticoid between severe respiratory infections group and non severe respiratory infections group (*P* < 0.05).

**Conclusions:**

This study demonstrated that FARP can provide the rapid detection of respiratory virus and atypical bacteria for children, especially with severe respiratory tract infections.

## Background

Respiratory tract infections (RTIs) including pneumonia, representing as the major infectious diseases in children with a high morbidity and mortality, are mainly caused by a series of bacteria and viruses [1, 2]. Previous estimates found that RTIs caused more than 2–6 million deaths worldwide in 2013, making them the fifth leading cause of death overall and the leading infectious cause of death in children younger than 5 years [3]. As is known to all, culture and antigen/antibody methods are conventional methods to detect infectious pathogens, but their low sensitivity and long turn-around time limits the application in clinical. Therefore, introduction of a rapid, sensitive, and specific diagnostic tool is urgently required to understand the epidemiological surveillance and clinical characteristics of RTIs. More recently, advances in polymerase chain reaction (PCR) techniques have aided in the rapid and accurate detection of infectious pathogens, which are beneficial for precise selection of therapeutic scheme in children [4–6].

The FilmArray respiratory panel (FARP) is a multiplexed, fully automated nested PCR assay, which can detect seventeen common respiratory virus and three atypical bacterial pathogens with a turnaround time of approximately 1 h [7]. Previous studies have shown that FARP assay reveals excellent clinical utility over the more traditional laboratory methods of virus culture and direct antigen tests [8–10]. Owing to the sensitive detection of respiratory viruses, more and more clinical laboratories have introduced this technique to solve intractable cases for clinicians. Data about FARP application in children is still unclear. The goals of the present study are to retrospectively describe the clinical performance of FARP in children with RTIs and further to characterize the clinical effect of FARP in children with severe conditions.

## Methods

### Study design and specimens

This study was conducted in a children specialized hospital between July 1st, 2017 and June 30th, 2018. Children from pediatric intensive care unit (PICU) were diagnosed with RTIs. The diagnostic criteria of respiratory infections is depicted as follows: (1) patient with or without fever (defined as body temperature ≥ 37.5 °C); (2) patient with at least one of the following clinical symptoms: cough, nasal obstruction, tachypnoea, nasal flaring, or hypoxia [11]. The etiology of RTIs of them could not be detected by conventional PCR and then the FARP assay for 17 pathogens was applied for further detection. Therefore, all of above children were enrolled into study. The diagnostic standard of severe RTIs should included at least with the following symptoms: poor conditions, unabting high fever, anorexia or dehydration, disturbance of consciousness, hypoxemia, cyanosis, dyspnea, radiological confirmation of multi-lobar involve, pleural effusion, extrapulmonary complications. This study was approved by the Ethics Committee of Shanghai Children’s Hospital. Written informed consent was obtained from the patients’ guardians on behalf of the children enrolled in this study.

According to the instruction, nasopharyngeal secretion (NPS) samples were collected on the basis of standard technique from these enrolled children by clinicians and immediately placed in viral transport media (VTM). Specimens in VTM should be processed and tested as soon as possible. If storage is required, specimens in VTM can be held at refrigerator temperature (2–8 °C) for up to 3 days. The FARP assay for 17 pathogens and routine microbiological methods including direct fluorescence assay (DFA) and culture method were simultaneously performed for the collected NPS samples.

### FARP assay

The FARP assay was performed by multiplex PCR according to the manufacturer’s instructions (BioMérieux, France) [12]. In short, 1 mLof hydration solution and 300 μl of NPS sample buffer were injected into the FilmArray pouch, respectively. Then the loaded pouch was placed into the FilmArray instrument, and a preprogrammed run was started. The procedure of FilmArray pouch included specimen extraction, nmPCR (nest multiplex PCR), and results interpretation. The following organism types and subtypes are identified: adenovirus, coronavirus 229E, coronavirus HKU1, coronavirus NL63, coronavirus OC43, human metapneumovirus, human rhinovirus/enterovirus, influenza A H1, influenza A H1 2009, influenza A H3, influenza B, parainfluenza virus 1, parainfluenza virus 2, parainfluenza virus 3, parainfluenza Virus 4, respiratory syncytial virus, *Bordetella pertussis*, *Chlamydophila pneumoniae*, and *Mycoplasma pneumoniae*. However, human rhinovirus and human enterovirus must be reported as indistinguishable since these they are closely related viruses and cross-positivity between those viruses is possible with the FARP assay [13].

### Other common methods

The eight viruses included adenovirus, influenza A, influenza B, parainfluenza Virus 1, parainfluenza virus 2, parainfluenza virus 3, respiratory syncytial virus, human metapneumovirus were commonly detected by direct fluorescence assay (DFA) according to the manufacturer’s instructions (Diagnostic hybrids, INC, USA). The antibody of *M.pneumoniae* was analyzed by passive particle agglutination and *B.pertussis* was analyzed by culture methods. Other pathogens were not identified in our clinical laboratory.

### Statistics analysis

All statistical analysis was performed by using SPSS 19.0 for Windows (version 22.0; SPSS Inc., Chicago, IL, USA). Clinical testing and the FARP assay were compared using the exact two-sided McNemar’s test. A value of *P* ≤ 0.05 was considered statistically significant.

## Results

### Clinical characteristic of enrolled patients

A total of ninety patients from PICU diagnosed with RTIs were enrolled into this study. The average age of all children was 2.55 ± 2.93 years, with 52 male and 38 female children. The children aged between 0 and 14 years were divided into four groups including infants (0–1 year, 34.4%), toddlers (1–2 years, 32.2%), preschoolers (3–5 years, 24.4%) and children (6–14 years, 8.9%). Moreover, 48 children (53.3%) were diagnosed with severe RTIs, and 46.7% of them were observed with underlying diseases including heart disease and intestinal diseases. Furthermore, a majority of children were improved after the treatment during hospitalization and 3 children were died. The general characteristics of these patients are presented in Table 1.

**Table 1:** Clinical characteristics of 90 children enrolled in this study

CharacCstics	All patients (%)	Severe respiratory infections group (%)	Non severe respiratory infection group (%)
No.	90	48	42
Gender			
Male	52 (57.80)	28 (58.3)	25 (59.52)
Female	38 (42.20)	20 (41.7)	17 (40.48)
Age			
<1 year	31 (34.44)	15 (31.25)	16 (38.10)
1-2 years	29 (32.22)	18 (37.50)	11 (26.19)
3-5 years	22 (24.44)	13 (27.08)	9 (21.43)
6-14 years	8 (8.89)	2 (4.17)	6 (14.29)
Hospitalization stay (days)	18.36±19.31	19.40±20.38	17.17±18.19
Cost (RMB)	61222.26±87707.67	73978.42±101649.49	46643.81±66676.26
Use of anti-virus (%)	20 (22.22)	17 (35.42)	3 (7.14)
Days of antibiotic use (d)	8.68±5.59	8.76±6.25	8.58±4.67
Use of glucocorticoid (%)	60 (66.67)	40 (83.33)	20 (47.62)
Underlying diseases			
None	62(68.89)	34 (70.83)	28 (66.67)
Heart diseases	9 (10.00)	4 (8.33)	5 (11.90)
Intestinal diseases	6 (6.67)	4 (8.33)	2 (4.76)
Central Nervous diseases	5 (5.56)	1 (2.08)	4 (9.52)
Other diseases	8 (8.89)	5 (10.42)	3 (7.14)
Clinical outcome			
Improved	74 (82.22)	37 (77.08)	37 (88.10)
Unhealed	13 (14.45)	9 (18.75)	4 (9.52)
Died	3 (3.33)	2 (4.17)	1 (2.38)

### Distribution of pathogens by FARP assay

Among 58 positive patients, 40 (67.0%, 40/58) patients had a single organism and 18 (31.0%, 18/58) patients had multiple organisms. Human rhinovirus/enterovirus was the most prevalent organism in 58 positive patients (29.3%, 17/58), followed by adenovirus (25.9, 15/58), parainfluenza virus 3 (19.0%, 11/58), respiratory syncytial virus (19.0%, 11/58). Other pathogens were as follows: *M.pneumoniae* (12.1%, 7/58), influenza A H1 2009 (8.6%, 5/58), human metapneumovirus (6.9%,4/58), influenza B (5.2%,3/58), *B.pertussis* (5.2%, 3/58), parainfluenza virus 1 (3.4%, 2/58), coronavirus HKU1 (1.7%,1/58), coronavirus NL63 (1.7%, 1/58), parainfluenza virus 4 (1.7%, 1/58) (Table 2). Several differences were detected among age. Higher proportions of various pathogens were identified in the infant and toddler (0–2 years) groups with human rhinovirus/enterovirus being mostly virus. Adenovirus were common in the group aged 3–5 years, but only three pathogens including *M.pneumoniae*, respiratory syncytial virus, and adenovirus were also found in age group (6–14 years).

**Table 2:** Distribution of all pathogens in children with respiratory infections

Pathogens	Total	Sigle positive group	Multi positive group	Severe respiratory infections group (48)	Non severe respiratory infection group (42)
Human Rhinovirius/Enterovirus	17	10	7	9	8
Adenovirus	15	7	8	10	5
Parainfluenza groups	14	6	8	8	6
1	2	1	1	1	1
2	0	0	0	0	0
3	11	4	7	6	5
4	1	1	0	1	0
Respiratory Syncytial Virus	11	5	6	6	5
*Mycoplasma pneumoniae*	7	2	5	5	2
Influenza A	5	2	3	4	1
H1 2009	5	2	3	4	1
H3	0	0	0	0	0
Human Metapneumovirus	4	2	2	2	2
Influenza B	3	2	1	3	0
*Bordetella pertussis*	3	1	2	0	3
Coronavirus groups	2	0	2	2	0
HKU1	1	0	1	1	0
NL63	1	0	1	1	0
229E	0	0	0	0	0
OC43	0	0	0	0	0
*Chlamydophila pneumonia*	0	0	0	0	0

The distribution of multi-organism combinations was depicted in Table 3. A total of 18 multi-organism children were detected with 13 various combination types. The combination of human rhinovirus/enterovirus plus parainfluenza virus 3 and adenovirus plus respiratory syncytial virus were the most common combination type. Additionally, the majority of multi-organism-positive patients were observed with adenovirus and human rhinovirus/enterovirus.

**Table 3:** Distribution of multi-organisms combinations in children with respiratory infections

Organism combination detected	No.
Human Rhinovirius/Enterovirus+Parainfluenza Virus 3	3
Adenovirus+Respiratory Syncytial Virus	3
Adenovirus+Parainfluenza Virus 3	2
Adenovirus+Influenza A H1 2009	1
Parainfluenza Virus 3+*Mycoplasma pneumoniae*	1
Human Rhinovirius/Enterovirus+*Mycoplasma pneumoniae*	1
Human Rhinovirius/Enteroviru+Human Metapneumovirus	1
Bordetella pertussis+Human Metapneumovirus	1
Bordetella pertussis+Human Rhinovirius/Enterovirus+Parainfluenza Virus 3	1
Adenovirus+Human Rhinovirius/Enterovirus+Respiratory Syncytial Virus	1
Adenovirus+Parainfluenza Virus 1+Coronavirus HKU1	1
Influenza A H1 2009+Respiratory Syncytial Virus+Influenza B	1
Influenza A H1 2009+Respiratory Syncytial Virus+Coronavirus NL63	1

### Comparison of FARP and other methods

The FARP assay was significantly more likely to detect a respiratory virus than DFA assay (*P* < 0.05). Among the ninety children, 58 children (64.4%) were identified with 13 pathogens by FARP assay, while only 11 (12.2%) children were detected with 5 viral pathogens (adenovirus, influenza A, respiratory syncytial virus, parainfluenza virus 1, and parainfluenza virus 3) by using DFA method. Among these 11 positive samples by DFA assay, 7 out of them were detected with more than 2 viral pathogens by FARP assay. Furthermore, the NPS samples were observed by FARP method within 1.7 h, which showed lower turnaround time (TAT) than DFA method with 5.2 h. Seven samples detected with *M.pneumoniae* by FARP analysis while only one samples were positive with *M.pneumoniae* antibody. Then three *B.pertussis* positive samples in FARP assay were negative in culture methods.

### Clinical significance of pathogens by FARP assay

The detailed clinical significance of 58 FARP positive children was showed in Table 4 and Table 5. Among 58 FARP positive children, 38 children (65.5%) were diagnosed with severe RTIs. According to the number of organisms detected, these children were divided into two groups including the single-organism-positive group and the multi-organism-positive group (Table 4). There was no significant difference in the length of hospitalization stay, hospitalization cost, use of anti-virus, rate of secondary infection, and clinical outcome (*P* > 0.05), while significant differences were observed for days of antibiotic use and usage of glucocorticoid between these two groups (*P* < 0.05). Furthermore, there was significant difference in use of anti-virus and usage of glucocorticoid between severe respiratory infections group and non severe respiratory infections group (*P* < 0.05) (Table 5).

**Table 4:** Comparison of clinical significance between multiple organisms and single organism group by FARP assay

Factors		Multiple organism group(40)	Single organism group(18)	T/X^**2**^	Value of ***P***
Hospitalization stay (days)		15.10±14.83	22.78±16.94	1.485	0.228
Cost (RMB)		56453.93±84859.31	66085.87±103706.31	0.007	0.932
Use of anti-virus (%)		11 (27.50)	9 (50.00)	2.782	0.095
Days of antibiotic use (d)		8.05±4.32	10.72±6.98	5.619	0.021
Use of glucocorticoid (%)		24 (60.00)	16 (88.90)	4.84	0.028
Secondary infection (%)		17 (42.50)	9 (50.00)	0.282	0.595
Clinical outcome	Improved	33 (82.50)	16 (88.89)	1.425	0.49
	Unhealed	4 (10.00)	2 (11.11)		
	Died	3 (7.50)	0

**Table 5:** Comparison of clinical significance in positive samples between severe respiratory infection group and non severe respiratory infection group

Factors		Severe respiratory infections group (38)	Non severe respiratory infections (20)	T/X2	Value of ***P***
Hospitalization stay (days)		18.89±14.87	14.80±17.46	0.892	0.741
Cost (RMB)		72381.71±98676.056	34859.89±67322.420	1.708	0.114
Use of anti-virus (%)		18 (47.37)	2 (10.00)	8.099	0.004
Days of antibiotic use (d)		8.82±5.72	9.00±4.78	0.13	0.764
Use of glucocorticoid (%)		32 (84.21)	8 (40.00)	11.966	0.001
Secondary infection (%		19 (50.00)	7 (35.00)	1.192	0.275
Clinical outcome	Improved	30 (78.95)	19 (95.00)		
	Unhealed	6 (15.79)	0	3.559	0.169
	Died	2 (5.26)	1 (5.00)		

## Discussion

Over the past decades, RTIs comprise as the most common diseases among children less than 5 years of age with the majority in low- and middle-income countries. The etiology of RTIs always contribute to virus and bacterial, including influenza, respiratory syncytial virus, *B.pertussis, S.pneumoniae,* and *H.influenzae* [14, 15]. In general, infrequent isolated pathogens were always found in several severe RTIs cases and viruses were considered as the leading cause, including adenovirus, respiratory syncytial virus, and metapneumovirus [16]. This study showed that human rhinovirus/enterovirus was the most common virus, especially in some patients where rhinovirus was the only virus identified. However, the role of rhinoviruses in serious respiratory infections remains controversial. Several researchers found that rhinovirus was the most prevalent virus in asymptomatic carriers with the rates ranging from 12 to 32%. A reviewer conducted by Jacobs SE et al. demonstrated that with the increasing implementation of PCR assays for respiratory virus detection in clinical practice, the recognition of rhinovirus as a lower respiratory tract pathogen had been facilitated, particularly in patients with asthma, infants, elderly patients, and immunocompromised hosts, and more data had emerged on the high incidence of rhinovirus infection, resulting in the further awareness of the widespread and sometimes serious disease manifestations [17]. Furthermore, respiratory viruses are responsible for bronchiolitis and pneumonia and can also lead to considerable economic burden in the terms of medical visit. In addition, atypical respiratory pathogens involving in *B.pertussiss, M. pneumoniae,* and *C.pneumoniae,* pose as the emerging respiratory pathogens and have become a health public problem in many countries. Several studies depict that clinical symptoms of atypical respiratory infections is indistinguishable from viral respiratory infections and that co-infection with other viruses also exist [18].

Recently, there has been an increasing interest that simultaneous infection with multiple pathogens is increasingly recognized as both common and important for disease manifestation. This study described that 18 children had more than two organisms, with human rhinovirus/enterovirus plus parainfluenza virus 3 and adenovirus plus respiratory syncytial virus being the most. It may make the treatment of simultaneous infections more difficult. Then co-infections between virus and bacterial isolates have been also detected in pediatric patients with RTIs [19] and this phenomenon was observed in 4 samples with *B.pertussis* or *M.pneumoniae* plus virus.

However, in regard to viruses and atypical organisms, it is truly difficult to detect by the traditional culture methods, owing to the long culture period and low sensitivity of these methods. FARP assay, a new technology, has been provided for detecting unidentified pathogens in respiratory samples. Previous studies demonstrated that FARP assay, which can simultaneously identify 17 viruses and 3 atypical organisms, showed high sensitivity and specificity than other route methods introduced in clinical [11, 20]. Our study demonstrated that in children with severe RTIs, the FARP assay has a higher positive detection rate than DFA assay available in our laboratory (64.4% vs 12.2%). Additional pathogens including coronavirus 229E, coronavirus HKU1, coronavirus NL63, coronavirus OC43, human rhinovirus/enterovirus, parainfluenza Virus 4, *B.pertussis*, *C.pneumoniae*, and *M.pneumoniae* were detected by FARP assay could not identified by DFA method.

Moreover, it is proposed that early diagnosis of pathogens in children with RTIs could decrease the length of hospitalization stay and reduce the mortality, especially for multiple organism infections. Generally, antibiotics have been commonly prescribed for many children with RTIs. While the samples were detected with positive pathogens by FARP assay within 1 h, the clinicians would immediately adjust the therapeutic schedule for children. According to the clinical data of these patients, we found that children identified with virus infections received or prolonged antivirus therapy and also reduced the inappropriate use of antibiotics during this process. A previous study reported that the mean duration of antibiotic use was significantly shorter after implementation of FARP assay than that before the implementation [21]. Furthermore, the length of hospitalization stay and hospitalization cost in the single-organism-positive group were still higher than these in the multi-organism-positive group, although there were no statistical difference in between these two groups.

## Conclusions

In conclusion, our study revealed that FARP assay can significantly detect respiratory virus and atypical bacteria in children, which could not be detected by conventional methods. Comparison of DFA assay, FARP assay can provide the rapid detection of a wide number of respiratory organisms within 1.7 h and especially render a valid choice for urgent pathogens in high-risk patients with severe respiratory infections. However, there still a limitation about the pathogen spectrum of FARP not including all pathogens. Therefore, combination of FARP and other molecular methods can make a significant improvement in diagnostic testing of respiratory pathogens.

## Data Availability

Please contact corresponding athor for data requests.

## Abbreviations

ARTIs: Acute respiratory tract infections
FARP: FilmArray respiratory panel
NPS: Nasopharyngeal secretion
PICU: Pediatric intensive care unit
PCR: Polymerase chain reaction
